# The Role of the COP9 Signalosome and Neddylation in DNA Damage Signaling and Repair

**DOI:** 10.3390/biom5042388

**Published:** 2015-09-30

**Authors:** Dudley Chung, Graham Dellaire

**Affiliations:** 1 Department of Pathology, Dalhousie University, Halifax, NS B3H 4R2, Canada; E-Mail: dudleyc@dal.ca; 2 Department of Biochemistry and Molecular Biology, Dalhousie University, Halifax, NS B3H 4R2, Canada

**Keywords:** COP9 Signalosome, DNA repair, NEDD8

## Abstract

The maintenance of genomic integrity is an important process in organisms as failure to sense and repair damaged DNA can result in a variety of diseases. Eukaryotic cells have developed complex DNA repair response (DDR) mechanisms to accurately sense and repair damaged DNA. Post-translational modifications by ubiquitin and ubiquitin-like proteins, such as SUMO and NEDD8, have roles in coordinating the progression of DDR. Proteins in the neddylation pathway have also been linked to regulating DDR. Of interest is the COP9 signalosome (CSN), a multi-subunit metalloprotease present in eukaryotes that removes NEDD8 from cullins and regulates the activity of cullin-RING ubiquitin ligases (CRLs). This in turn regulates the stability and turnover of a host of CRL-targeted proteins, some of which have established roles in DDR. This review will summarize the current knowledge on the role of the CSN and neddylation in DNA repair.

## 1. Introduction

### 1.1. The COP9 Signalosome

The COP9 Signalosome (CSN) is a multi-subunit protein complex that was identified in the 1990s in *Arabidopsis* as a repressor of photomorphogenesis [1], and was later found conserved in other unicellular and multicellular eukaryotes [2,3,4,5,6,7]. In eukaryotes that have simpler CSN complexes, such as yeast, several subunit deletions are viable [8,9,10]. However, null deletions in other organisms are lethal early in development [11,12,13], and conditional knockouts result in developmental phenotypes and impaired cellular functional [14], suggesting an increase in functional complexity as the CSN evolved. The role of the CSN is to deneddylate substrates, particularly cullin-RING E3 ubiquitin ligases (CRLs) in the ubiquitin proteasome pathway [15,16]. In addition, early attempts to biochemically isolate and characterize the CSN protein complex found it to be associated with kinase activity [2], which the molecule curcumin was able to inhibit [17]. Later studies identified the kinases that interact with the CSN to impart the complex with associated kinase activity. Examples include protein kinase CK2 (CK2) [18,19], protein kinase D (PKD) ([18], protein kinase B-Akt (Akt) [19], ataxia telangiectasia mutated (ATM)[20], and inositol 1,3,4-triphosphate 5/6 kinase [21]. These kinases modify the stability of ubiquitin-mediated proteasomal substrates. Since its discovery, researchers have begun to uncover roles for the CSN and the neddylation pathway in the DNA damage response (DDR). This review will explore the molecular mechanism of the CSN and current knowledge of its role in DNA damage signaling and repair.

### 1.2. CSN Architecture and Expression

The mammalian CSN holoenzyme consists of eight subunits (CSN1 to CSN8) [2,4]. Six of the eight subunits (CSN1-4, and CSN7-8) contain a PCI (proteasome, COP9, initiation factor) domain, a feature shared with subunits of both the 19S proteasome regulatory complex and eiF3 complex, suggesting a common evolutionary origin [17,22]. Furthermore, studies suggest these complexes can interact with one another [4,22,23,24]. CSN5, which is also called Jun activation domain-binding protein-1 (Jab1) [25], and CSN6 both contain an MPN (MPR1-PAD1-amino terminal) domain [26]. Unlike CSN6, the MPN domain in CSN5 contains a Zn^2+^ binding JAMM (JAB1/MPN/Mov34) motif, thus making it the sole catalytically active subunit in the CSN [10]. The metalloprotease JAMM/MPN motif possesses the His-X-His-X10-Asp consensus sequence (where X indicates any amino acid residue) accompanied by a conserved glutamic acid upstream [26]. In addition, mammals express two forms of CSN7 (CSN7a and CSN7b) and CSN complexes likely contain either one or the other of these two isoforms [27].

**Figure 1:**
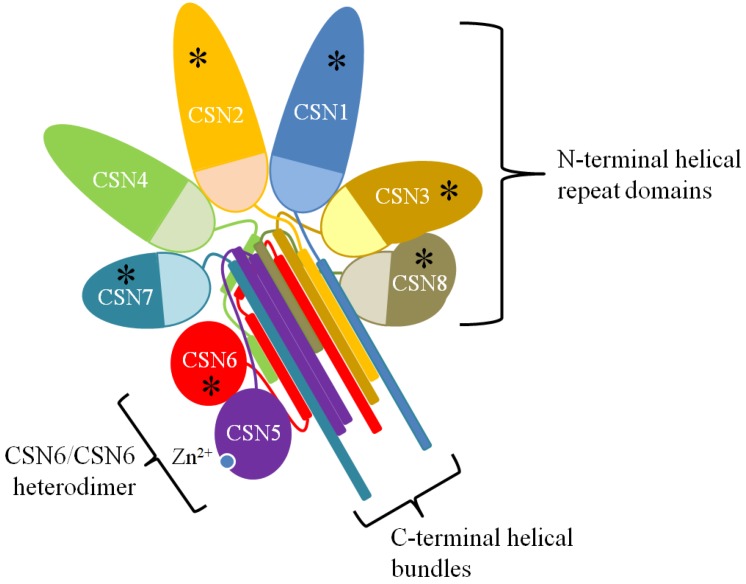
The CSN Structure. A two-dimensional schematic representation of the three-dimensional structure of the CSN as determined by Lingaraju *et al* [34]. The N-terminal repeat domains radiate out from the winged-helix domains of the PCI ring (lightly shaded half-circles). The C-terminal helical regions form a helical bundle that stabilizes the complex. The MPN domains of CSN5 and CSN6 rest on the helical bundle. Subunits reported as phosphorylation targets are marked with an asterisk (*).

Recent investigation of the individual subunits and of the CSN holoenzyme provided new details to its organization [28,29,30,31,32,33,34] (Figure 1). Current understanding is that the winged-helix domains of the PCI domains (PCI ring) of CSN1-4 and CSN7-8 are arranged as an open ring such that the N-terminal helical repeat domains of these subunits radiate out from it while the carboxy terminal helical tails form a bundle that anchor the complex [31,33,34,35]. The MPN domains of the CSN5-CSN6 heterodimer rest on the helical bundle while their carboxy terminal helical tails are inserted into the helical bundle. Integration of CSN5 into the complex is abrogated by the absence of CSN6, but deleting CSN1, 2, 4, or 7 can also disfavor CSN5 integration [34]. CSN4 and CSN6 appear to be the most important for stabilizing and converting CSN5 into its active state, which was recently found to involve rearrangement within CSN5 to open the NEDD8 binding pocket [11,34,36] but full enzymatic activity *in vitro* requires the complete set of subunits [29]. The peripheral association of CSN5 with the complex is dynamic since free/monomeric CSN5 is a feature found in different organisms. However, evidence suggests that free CSN5 is essentially catalytically inactive [11,26,29,36,37,38,39]. Nonetheless, one cannot rule out an as yet to be identified non-catalytic role for free CSN5 in the cell.

The CSN is catalytically active in both the nuclear and cytoplasmic fractions [40,41,42,43,44]. Additionally, a small fraction of CSN is bound to chromatin [37,45]. The CSN can be post-translationally modified, and, indeed, several subunits contain phosphorylation sites [17,20,37,46,47,48,49,50,51]. As a consequence, different cellular compartments can harbour different post-translationally modified CSN, and a great deal of work remains to understand the regulation of CSN subunits through their phosphorylation [37].

### 1.3. Neddylation Cascade

**Figure 2:**
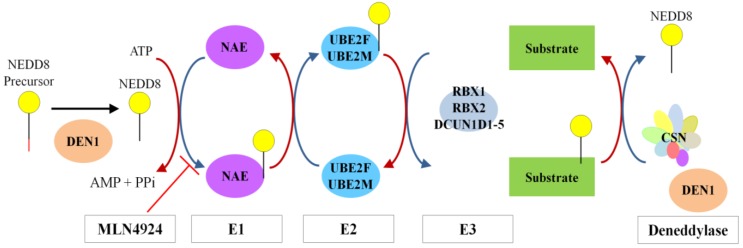
The Neddylation Cascade. A schematic representation of the main proteins in the neddylation cascade. Precursor NEDD8 is processed at the C-terminal to the activated form by DEN1. NEDD8 is conjugated to the target substrate through an E1 (NAE), and E2 (UBE2F or UBE2M), and an E3 (shown are RBX1/2, RNF111 and DCUN1D members). Deneddylation is achieved by the CSN and DEN1. The small molecule MLN4924 inhibits NAE, blocking the cascade.

Neddylation is a form of reversible post-translational modification whereby the ubiquitin-like protein NEDD8 (neural precursor cell expressed, developmentally down-regulated 8) is conjugated to lysine residues in the target protein. Expression of the NEDD8 gene was initially identified to be downregulated during mouse brain development [52], and encodes an 81-amino acid protein that is 60% identical and 80% homologous to ubiquitin [53,54]. The neddylation pathway consists of E1, E2, and E3 enzymes, analogous to the ubiquitylation pathway (Figure 2). Precursor NEDD8 is processed at the C-terminal tail (Gly76) to its mature form by deneddylase 1 (DEN1), also known as NEDP1 or SENP8 [55,56,57], and by ubiquitin C-terminal hydrolase isozyme 3 (UCHL3) [58]. Matured NEDD8, with its C-terminal gly-gly motif, is conjugated to the NEDD8 E1 activating enzyme (NAE), a heterodimer composed of amyloid-β precursor protein binding protein 1 (APPBP1, alternatively named NAE1) and ubiquitin-activating enzyme 3 (UBA3) [59,60]. NAE then transfers NEDD8 to an E2, which in metazoans are ubiquitin-conjugating enzyme E2F (UBE2F) and ubiquitin-conjugating enzyme E2M (UBE2M, also known as UBC12) [61,62]. UBE2F or UBE2M, with assistance from an E3, then transfers the NEDD8 onto the lysine of the target substrate.

Only a few E3s have been described to aid in neddylating targets. RING box protein 1 (RBX1, also known as ROC1) interacting with UBE2M, and RBX2 (ROC2) interacting with UBE2F, are E3s for cullin-RING ubiquitin ligases [16,63,64,65]. Neddylation E3 activity have also been described for ring finger protein 111 (RNF111-Arkadia) [66], and defective in cullin neddylation 1 domain containing (DCUN1D) proteins DCUN1D1-5 (SCCRO1-5) [67,68,69,70,71]. While DCUN1D1 is not essential for neddylation *in vitro* [72], DCUN1D1 knockouts are lethal in yeast and *Caenorhabditis elegans* [67]. However, this is not the case in mice, possibly due to compensation by other DCUN1D members [73]. Although it has been assumed that the DCUN1D proteins play similar roles in promoting neddylation, the case is not so clear for DCUN1D3 (SCCRO3). In one study, DCUN1D3 was shown to interact with UBE2M and promote cullin neddylation [70]. However, a later study found that DCUN1D3 does not have E3 activity and can inhibit DCUN1D1-mediated neddylation [73]. Additional proteins that exhibit NEDD8 E3 activity include murine double minute 2 (MDM2) [74], c-CBL [75,76], yeast Tfb3 [77], tripartite motif containing 40 (TRIM40) [78], and SMAD specific E3 ubiquitin protein ligase 1 (SMURF1) [79].

Although both the CSN and DEN1 can theoretically deneddylate a given protein substrate, they in fact do not have extensively overlapping protein targets [26,55,80]. DEN1 is more efficient in deconjugating hyperneddylated cullins in comparison to mono-neddylated CRLs *in vitro*, and DEN1 can deconjugate NEDD8 from non-cullin proteins *in vivo* in plants and humans [57,81]. On the other hand, the CSN appears restricted to deconjugating mono-neddylated substrates and is not efficient in processing precursor NEDD8 [57]. Recent evidence also indicates that the CSN can regulate human DEN1 and *Aspergillus nidulans* homolog DenA protein levels; however, the exact regulatory mechanism remains unknown [82]. Thus, the potential for cross-talk between these deneddylating enzymes should be carefully considered when interpreting CSN knock-down studies.

### 1.4. Regulation of Cullins by the CSN

The majority of proteins in the cell, including proteins in the DNA damage response, are targeted by different families of ubiquitin ligases. The multi-subunit cullin-RING ubiquitin ligases (CRLs) comprise the largest class of ligases [16]. The basic structure of the CRL consists of the cullin protein and the RING finger protein, which brings the substrate and substrate-specific adaptors in close proximity to the ubiquitin-carrying E2 protein; therefore, facilitating the transfer of ubiquitin onto the lysine residue on the target. CRLs are classed based on the cullin scaffold protein (CUL1-5, and CUL7), and specificity is defined by the cullin and a multitude of substrate adaptor proteins. (See [16,83] for additional background on CRLs). All cullins are modified by NEDD8 [84,85]. A more complex picture of the regulation of the CRL is emerging and a few regulatory mechanisms have been described thus far. As a result, this section will primarily discuss the role the CSN has on regulating CRLs.

Activation of the CRL is understood to be through the covalent attachment of NEDD8 to cullins, which as mentioned previously is mediated by RBX1 and DCUN1D1 members [64]. This causes a conformational change in the CRL architecture, promoting assembly, and enables substrate ubiquitylation [84]. For example, one study using CUL5^CTD^-RBX1 found that the neddylation led to a reorientation of the RING finger protein RBX1 [86]. Researchers have identified MLN4924 as a specific inhibitor of the E1 component APPBP1 (NAE1) [87]. This drug, which mimics the structure of AMP, forms an adduct with NEDD8 via NAE (Figure 2), blocking the neddylation cascade which leads to a reduction in neddylated CRLs and CRL substrate accumulation in cells [88]. This demonstrates that neddylation strongly regulates CRL-mediated ubquitylation and/or turnover of protein substrates.

**Figure 3:**
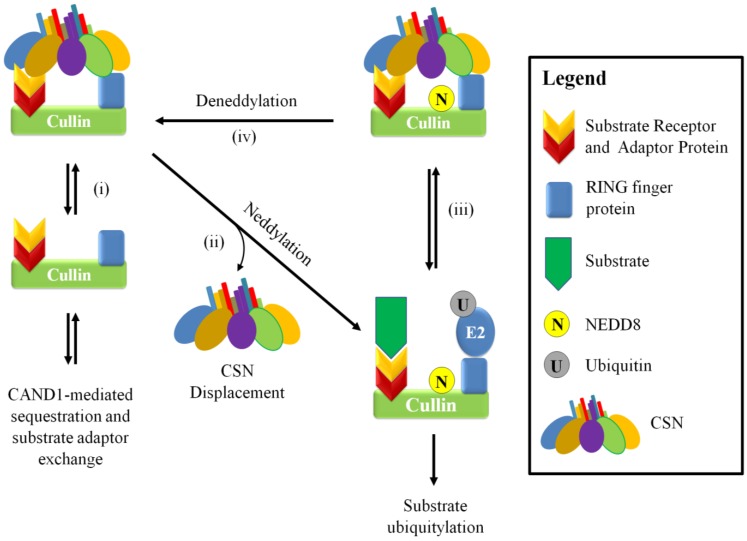
A schematic model for the neddylation-dependent regulation of CRLs by the CSN. The CSN can bind and inhibit substrate ubiquitylation in a neddylation dependent manner. Deneddylated CRLs can be a substrate for CAND1-mediated cullin sequestration and substrate adaptor exchange (i), but it can be activated through neddylation and CSN displacement to promote substrate and E2 binding and subsequent substrate ubiquitylation (ii). Interaction between the CRL to the CSN (iii and Figure 5) positions and activates CSN5 to allow deneddylation to occur (iv).

The deneddylation of CRLs is achieved by the CSN, through its catalytic subunit CSN5. Initial understanding was that the CSN holoenzyme is transiently associated with the CRL to deneddylate cullins; however, a more complex picture of its role has begun to emerge. Structural analysis of the CSN-CRL association suggests that *in vitro* interaction with various cullins can further promote CSN5 activation [34,89]. The deneddylated cullin is a substrate for the protein CAND1 (cullin-associated NEDD8-dissociated protein 1), which regulates CRL activity by sequestering deneddylated cullins [90,91] (Figure 3). However, this interaction can be reversed depending on the levels of substrate adaptor proteins. CAND1 regulation was shown to only affect deneddylated CRLs, since adding CAND1 to assembled CRLs containing neddylated cullin blocked substrate adaptor dissociation [92]. The current belief is that that CAND1 promotes exchanging of the substrate adaptors in response to changing conditions in the cell [92,93,94,95]. It should be noted that CAND1 does not associate to the same degree with different cullin classes and may also display a preference to the exchange of particular substrate adaptors [95].

**Figure 4:**
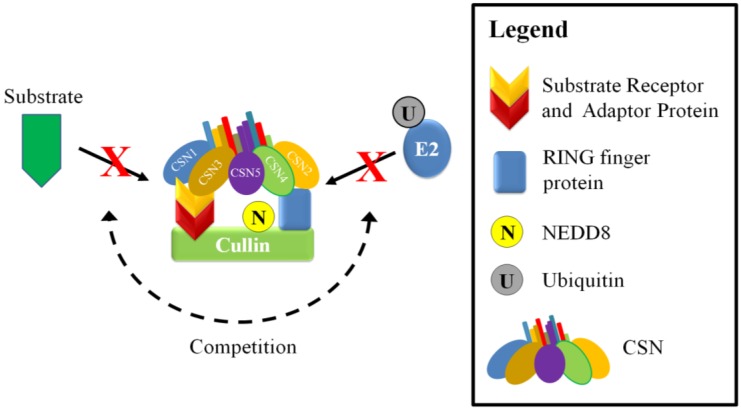
A schematic model of neddylation-independent regulation of CRLs by the CSN. The CSN interaction can inhibit CRLs in a neddylation independent manner by competing with substrates and ubiquitin-E2s for binding sites.

**Figure 5:**
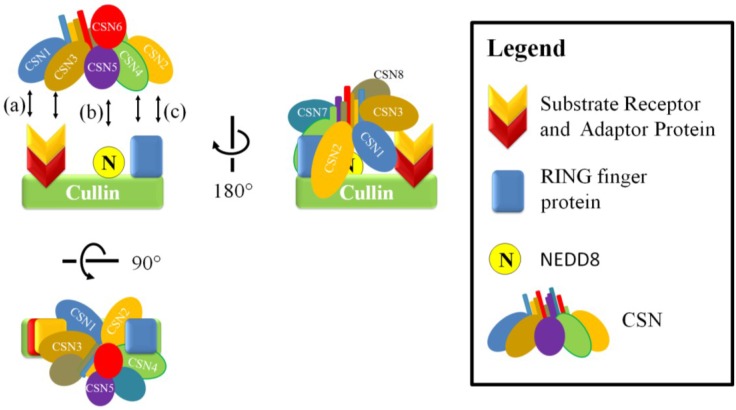
The CSN and CRL interaction. The CSN-CRL association involves the interaction of CSN1 and CSN3 on the substrate receptor (a) and CSN2 and CSN4 on the RING finger protein and the C-terminal portion of the cullin (c) [34,89]. These interactions position and activate CSN5 to allow deneddylation to occur (b).

The CSN can also inhibit CRL activity independently of its deneddylase activity. The CSN can bind directly to CRLs and reduce ubiquitin ligase activity by sterically hindering interaction between the target substrate and the E2 (Figure 4) [89,96]. It appears that this mode of regulation can be influenced by the levels of substrate, which can compete with the CSN for the cullin [89,97]. This was demonstrated in one study where there was a reduction of CSN-CRL association when preincubated CSN-CRL was placed in the presence of substrate [89,96,97]. Additionally, global mass spectrometry studies on the cullin proteins found that on average only 10%–20% are associated with the CSN; whereas the association with substrate adaptors was dominant, suggesting that substrate adaptor availability is important in regulating CRLs [95]. The CSN is able to associate with the cullin in both neddylated and unneddylated states. In a study focusing on the cullin 1 CRL, SCF^SKP2/CKS1^, CSN2 and CSN4 appear to be important in the interaction with the cullin and RING finger protein; whereas the other subunits, such as CSN1 and CSN3, are oriented toward the substrate adaptors (Figure 5) [34,89]. Association of the CSN to CRLs does not immediately lead to deneddylation. In a study that used o-ophenathroline to inhibit deneddylation after cell lysis, up to half the CSN-associated cullins were also neddylated [95]. This may indicate that an additional signal is required to allow isopeptidase cleavage or that the CSN is inhibited by some unknown factor, such as a CRL architecture that disfavors CSN-mediated deneddylation.

In addition to direct deneddylation, and steric hindrance, the CSN can associate with the de-ubiquitylating enzyme USP15. For example, in the fission yeast *Schizosaccharomyces pombe*, the CSN associates with USP15 homolog Ubp12p [98] to inhibit ubiquitylation of substrates and autoubiquitylation of CRL components [99]. Therefore, it is thought that ubiquitylation takes place after the CSN is displaced from the CRL complex.

## 2. CSN and Neddylation in the DNA Damage Response

### 2.1. The DNA Damage Response

Organisms have evolved complex systems that form the DNA damage response (DDR) to protect their genome from unwanted damage. These pathways sense and recognize different types of damage, and signal the activation of proteins for appropriate repair of DNA lesions. Since DNA damage comes in many forms, each type activates a unique repair response. Endogenous sources of DNA damage include hydrolysis (deamination, depurination, and depyrimidination), alkylation (6-O-Methylguanine) and oxidation (8-oxoG) by reactive oxygen species generated by respiration, and DNA mismatches during replication [100,101]. Exogenous sources of DNA damage include physical (ionizing radiation (IR), ultraviolet light (UV)) and chemical (such as chemotherapeutic drugs, and environmental carcinogens like industrial chemicals and tobacco smoke) [101]. The type of damage can be covalent modifications, single-strand and double-strand DNA breaks. In human cells, there are many forms of DNA repair, but not all of which have been associated with the CSN. However, nucleotide excision repair (NER) and double-strand break (DSB) repair have the most compelling data implicating the CSN in these mechanisms, which will be described in detail below.

Despite the form of DNA damage and specific repair mechanism involved, all have a defined hierarchy of protein recruitment. The initial response begins with the recruitment of proteins that recognize the damage or alteration to DNA (“sensors”), followed by those that receive the signal from the sensors and transmit it downstream (“mediators/transducers”), ultimately recruiting proteins that repair the lesion (“effectors”). Cytologically, these proteins form observable nuclear foci, and the number of foci corresponds to the degree of DNA damage [102]. Depending on the severity of the damage, a number of cellular changes occur including chromatin reorganization and alterations in transcription activity, and the activation of checkpoints to delay or stop cell cycle progression, senescence, and apoptosis [101]. Despite the many variations in the systems under the DDR umbrella, a common theme is the importance of ubiquitin and ubiquitin-like proteins and their corresponding E3 ubiquitin ligases. Many studies have characterized complex and overlapping modifications by ubiquitin-like proteins in damage sensing and mediating proteins as well as chromatin binding proteins [103]. This suggests that the CSN and neddylation could be important for regulating the DDR.

### 2.2. The CSN and Neddylation in the DNA Damage Response

In response to DNA damage, multiple processes such as cell cycle progression and checkpoint control are activated and involve the CSN and neddylation through CRLs [5,104]. For example, through ectopically expressing individual CSN subunits, it was found that CSN5 binds to the cyclin-dependent kinase inhibitor p27^Kip1^ and targets it for ubiquitin-mediated proteasomal degradation in the G1 cell cycle phase [105]. This shuttling of p27^Kip1^ from the nucleus to the cytoplasm could be mediated by additional interaction with CSN6 and the E3 ubiquitin ligase constitutive photomorphogenic 1 (COP1) [106]. Additionally, p27 degradation was observed from S to G2 phase, which involved p27 interaction with the CSN and CK2, and phosphorylation by CK2 [19]. Chromatin licensing and DNA replication factor 1 (CDT1), which is important for initiating DNA synthesis during S-phase in undamaged cells, is ubiquitylated by CRL4 [104]. The effect of neddylation in cell cycle regulation becomes apparent when cells are treated with MLN4924. These cells have altered S-phase progression in the cell cycle due to inhibition of CRL1^SKP2^ and CRL4^CDT2^, which stabilizes CDT1 leading to additional rounds of DNA replication in S phase cells [107]. Other CRL-regulated cell cycle proteins, including WEE1 and cyclin-dependent kinase 2 (CDK2), also accumulate in the presence of MLN4924 [108].

The CSN has a direct and indirect role in p53 regulation. The p53 protein is important for regulating cell fate in response to diverse cellular stress including DNA damage, which typically results in upregulation of p53 expression in response to genotoxic stress. Increased levels of p53 will, in turn, upregulate expression of the kinase inhibitor p21. Because p21 binds to CDK2 and inhibits its activity, the cell arrests at G1/S to prevent DNA replication until DNA damage is repaired [109]. In addition to activating cell cycle checkpoints, p53 regulates additional DDR responses such as senescence and apoptosis [110]. Early investigations found that specific phosphorylation of p53 on Threonine 155 promoted degradation through its interaction with MDM2. This phosphorylation appeared to be mediated by the p53 interaction with CSN5 in the holoenzyme [21,111]. In addition, MDM2 and CSN5 can regulate the export of p53 from the nucleus into the cytoplasm for degradation [112]. Similarly, it was found that over-expression of CSN6 can promote p53 degradation through inhibiting autoubiquitylation of MDM2, and mice that were heterozygous for *CSN6* were more susceptible to DNA damage [113]. HER2-Akt signaling may also promote p53 degradation by promoting the stability of CSN6 in addition to phosphorylation and stabilization of MDM2 [51,114]. The p53 protein is also reported to be neddylated by MDM2 as well the SKP1-cullin-F-box (SCF) E3 ligase complex containing FBXO11 [74,115]. Currently, the biological role for p53 neddylation is not well characterized but is believed to impact p53 transcriptional activity [74,115]. The p53 gene (*i.e.*, *TP53*) itself is also indirectly regulated via neddylation of the ribosomal protein L11, which is found in the nucleolus conjugated to NEDD8 in unstressed cells [116]. DNA damage is able to disrupt the nucleolus, which releases L11 into the nucleoplasm [117,118,119]. Nucleoplasmic L11 is then deneddylated, possibly by DEN1, which allow L11 to be recruited to the *TP53* promoter [116,117]. The localization of L11 was also recently found to be regulated by the protein Myeloma overexpressed 2 (Myeov2). Myeov2 can sequester L11 in the nucleoplasm and promotes deneddylation of a host of proteins including L11, which in turn would impact *TP53* gene expression [116]. Interestingly Myeov2 also interacts with the CSN holoenzyme via interaction with CSN5, and while this interaction does not appear to affect L11 neddylation in the experimental conditions used, it raises the possibility for a neddylation-dependent role in either nucleolar maintenance and/or *TP53* gene regulation [116]. Finally, p53 transcriptional targets have also been shown to be regulated through neddylation. For example, the stability of the p53-regulated protein 14-3-3σ, a cell cycle regulator, appears to be regulated through interactions with CSN6 and COP1 [120].

### 2.3. Nucleotide Excision Repair

Helix-distorting forms of DNA damage are repaired by nucleotide excision repair (NER) [121]. Examples of these are cyclobutane pyrimidine dimers (CPDs) and pyrimidine 6-4 pyrimidone photoproducts (6-4PPs) created from ultraviolet (UV) radiation, and bulky adducts generated from environmental mutagens and chemotherapeutic drugs. There are two main types of NER: global genomic NER (GG-NER) that works on the whole genome, and transcription-coupled NER (TC-NER), which is restricted to actively transcribing genes. Impaired NER can contribute to genetic diseases. Two well-known hereditary photosensitivity diseases are Cockayne syndrome and xeroderma pigmantosum (XP). XP patients have an over 2000-fold increase risk of developing skin cancer because they cannot repair UV-induced lesions [122].

For GG-NER, mildly distorting lesions, such as CPDs and 6-4PPs, are most likely sensed by damaged DNA binding protein 2 (DDB2, also known as p48 or XPE), whereas larger distorting lesions are likely sensed by XPC-RAD23 [96,123,124] (see [121,125,126] for comprehensive reviews on the NER pathway). Briefly, after encountering a CPD or 6-4PP, CRL4^DDB2^ ubiquitylates XPC. Binding of XPC allows other GG-NER proteins to be recruited to the site, such as transciption factor II Human (TFIIH). TFIIH, a multi-subunit complex with 3'–5' and 5'–3' helicase activity, is recruited to unwind the DNA at the damage site to form a repair bubble. XPC leaves and TFIIH recruits XPG, which incises the DNA backbone 3' to the lesion. XPA can interact with other NER proteins and is thought to coordinate the steps in NER, and replication protein A (RPA) positions XPC and XPF•ERCC1 (excision repair cross-complementation group 1) on the DNA [127]. XPF•ERCC1 binds to the complex and incises the DNA backbone 5' to the lesion. Several members of the complex and the cut oligonucleotide dissociate, and DNA synthesis proteins RFC (replication factor C)/PCNA (proliferating cell nuclear antigen) and DNA polymerase δ/ε fills in the gap that is then sealed by DNA ligase 1.

TC-NER involves CSA (Cockayne syndrome WD repeat protein A, also known as ERCC8) and CSB (Cockayne syndrome protein B, also known as ERCC6) instead of XPC and DDB2 [128]. When the RNA polymerase encounters the lesion, it stalls, which is a damage recognition signal for repair. The stalled RNA polymerase II (RNAPII) then complexes with CSB to facilitate recruitment of NER components. Meanwhile, CSA forms an active complex with cullin 4 and DDB2 (CRL4^CSA^) [45]. Following the recruitment of NER components, such as TFIIH, RPA and XPA, repair proceeds in the same manner as described for GG-NER.

### 2.4. Cullin 4 E3 Ubiquitin Ligase and NER

**Figure 6:**
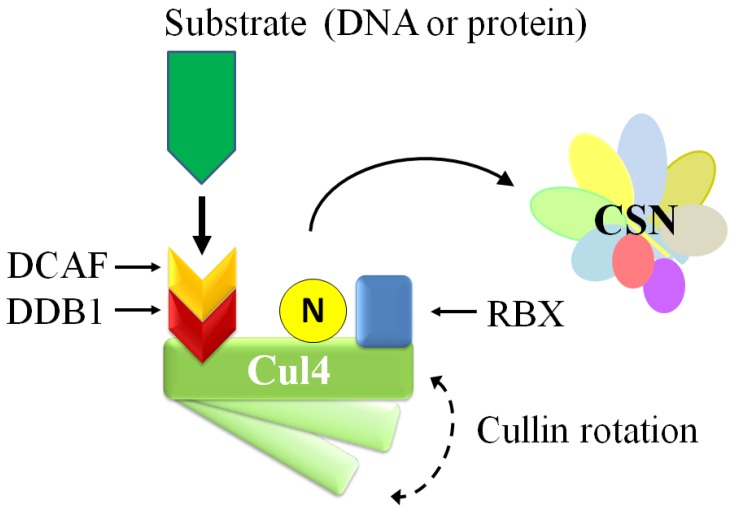
The architecture and activation of CRL4. The CRL4 architecture (adapted from Fischer *et al.* [96]) is composed of the scaffold protein cullin 4, RBX1, DDB1, and a family of substrate-specific receptor proteins termed DCAFs (Ddb1- and Cul4-associated factors), which DDB2 and CSA are members. Upon binding to CPDs or 6-4PPs on the DNA strand via DDB2, or a protein target (e.g., CSB via CSA), CRL4 undergoes a conformation change that promotes CSN displacement and neddylation to activate the ligase.

Discussion of the role of the CSN and neddylation in NER requires a brief introduction to CRL4. The CRL4 architecture is composed of the scaffold protein cullin 4, RBX1, DDB1, and a family of substrate-specific receptor proteins termed DCAFs (D*db1*- and C*ul4*-associated factors) (Figure 6) [96]. Cullin 4 exists as two related forms in humans and other higher eukaryotes. Because of their high sequence identity, and that their respective CRL architectures are nearly identical, cullin 4A and cullin 4B share functional redundancy with limited exceptions [96,129]. Cullin 4 is involved in different aspects of the DNA damage response. Because untranscribed DNA is packaged more tightly into chromatin, the repair proteins have to gain access to (UV-induced) DNA damage sites. One approach is to modify histone proteins, and another is to remodel chromatin. Of note is the role of DDB2, which forms a heterodimer with DDB1 (p127) in the DDB complex. DDB1/2 associates with CRL4s to bring cullin 4 to chromatin after UV-mediated DNA damage. DDB2 probes the DNA strand for damage, and when detected it exposes, and stabilizes the CPD and 6-4PP lesions. Structural and molecular analysis indicates that this is mediated by the β-hairpin and WD40 domains within DDB2 [96,123]. Compared to XPC, DDB2 has a higher affinity for CPDs and 6-4PPs. This DNA interaction leads to CSN displacement, CRL4^DDB2^ reconfiguration, neddylation, and ligase activation, which allows the ubiquitylation of XPC, histones, and possibly other proteins in a zone surrounding the lesion [123,124]. DDB2 itself is a target of CRL4 [130,131] and ubiquitylated DDB2 is thought to allow dissociation from the repair site. CRL4^DDB2^ ubiquitin ligases can also serve to ubiquitylate histones around the damage site *in vitro* to promote the recruitment of NER proteins such as XPA [132]; however, further studies are needed to fully understand its role [123]. In addition to affecting chromatin remodeling, XPC polyubiquitylation by CRL4 improves its stability and ability to bind to DNA, but so far the precise ubiquitylation site is unknown [124]. Instead, XPC eventually dissociates from the repair site to allow binding of downstream proteins. Recent investigations have revealed the possible role of an additional E3 ligase in this event. The SUMO-targeted ubiquitin ligase (STUbL) RNF111 ubiquitylates SUMOylated XPC at the residue K63 and is thought to promote dissociation from the damage site and association of downstream proteins XPG and ERCC1 [133,134,135]. In addition, polyubquitylated XPC is not immediately degraded by the 26S proteasome because its association with RAD23, a protein with ubiquitin-associated (UBA) domains, inhibits its degradation until XPC has bound to damaged DNA [136,137,138,139,140].

In TC-NER, cullin 4 forms an active complex with CSA and DDB2 (CRL4^CSA^) that is constitutively associatedd with chromatin [45]. The role of CRL4^CSA^ is to possibly ubiquitylate the stalled and hyperphosphorylated RNAPII [141,142]. CRL4 was found to target CSB for ubiquitylation and degradation several hours after UV irradiation in HeLa cells, suggesting a model where CRL4 is important for terminating the TC-NER response [130]. However, this was not observed in another study using a different cell line [131]. Other studies have also identified a UVSSA (UV-stimulated scaffold protein A)-mediated recruitment of the deubiquitylating enzyme ubiquitin-specific peptidase 7 (USP7), which can counteract CSB ubiquitylation [143,144]. Despite these recent insights, much remains to be uncovered about the role CRL4 plays in TC-NER.

### 2.5. The CSN and NER

**Figure 7:**
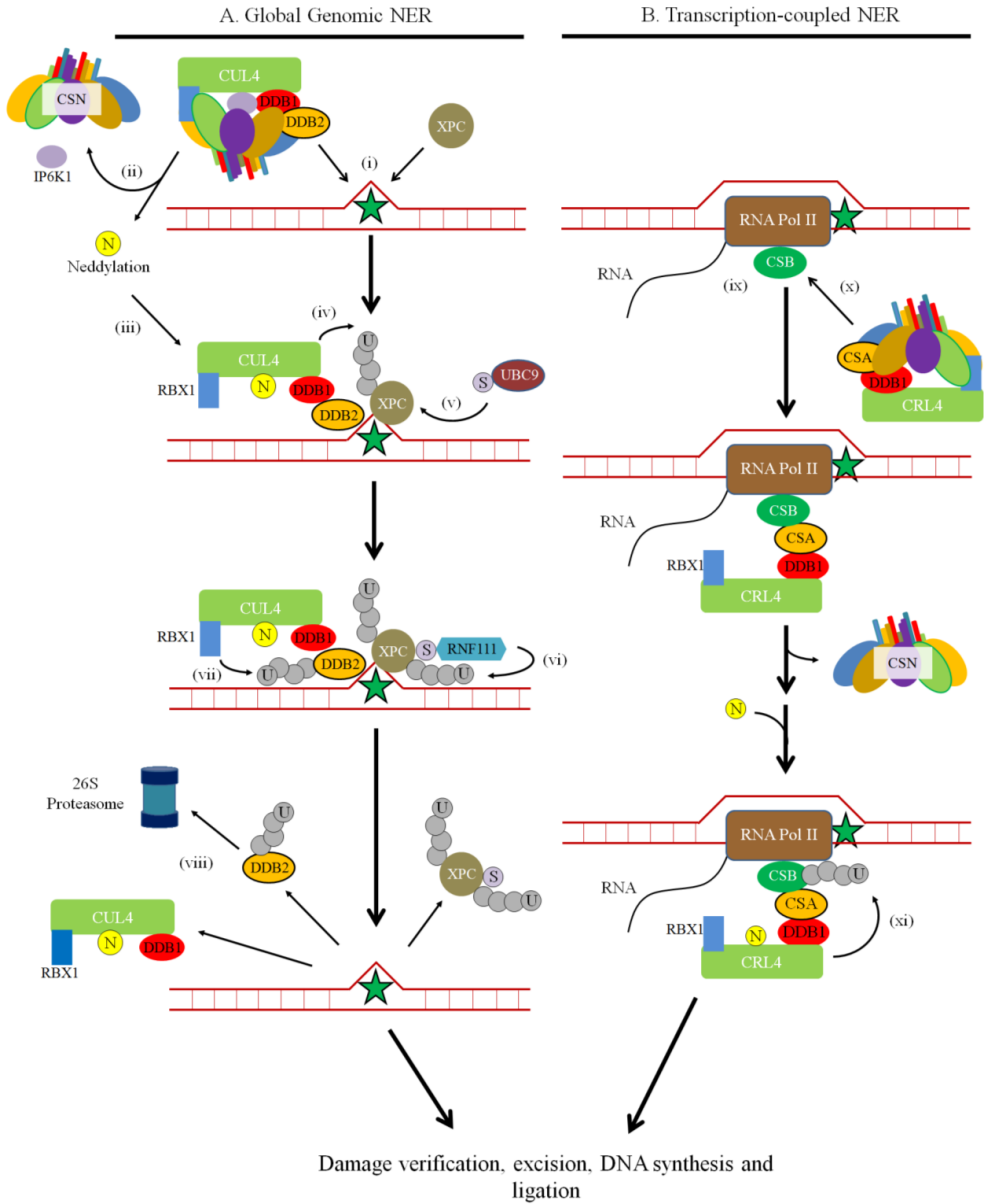
The Role of the CSN and Neddylation in the Initial Steps of NER. Cells use nucleotide excision repair to remove bulky lesions on DNA. (**A**) In GG-NER, XPC-RAD23 or DDB1-DDB2 recognizes the lesions (indicated by the star) (i). The CSN dissociates from CRL4 with the assistance of IP6K1 (ii), which is thought to promote NEDD8 (N) conjugation and CRL4 activation (iii). DDB1-DDB2 forms a complex with CUL4 and RBX1 to ubiquitylate XPC, which recruit repair proteins involved in subsequent steps in GG-NER (iv). XPC is SUMOylated by UBC9 (v), which promote ubiquitylation at Lys63 by RNF111 (vi) and dissociation from the DNA. Additionally, CRL4-DDB1 ubiquitylates DDB2 (vii) to promote its degradation by the 26S proteasome (viii). (**B**) In TC-NER, CSB associates to the stalled RNA Polymerase II (RNA Pol II) (ix). Through CSA, CRL4 is recruited to CSB (x). Following CSN dissociation and neddylation, CRL4^CSA^ possibly ubiquitylates CSB (xi). Both the GG-NER and TC-NER repair pathways then proceed with damage verification, damaged nucleotide excision, DNA synthesis, and ligation. U = Ubiquitin. S = SUMO protein.

The CSN regulates NER through CRL4^DDB2^ and CRL4^CSA^. CSN associates with CRL4^DDB2^ to regulate it through deneddylation or accessibility of binding surfaces (Figure 7). In untreated cells, the CSN is associated with DDB2 complexes in the purified chromatin fraction. However, UV irradiation increases CRL4 neddylation and the association with CSN is no longer present in the chromatin fraction [45]. The question of what causes the CSN to dissociate from CRL4^DDB2^ after UV damage was addressed Rao *et al.* [145], who found that it is facilitated by inositol hexakisphosphate kinase 1 (IP6K1). IP6K1 is an interacting partner with CSN1, CSN2 and DDB1, but is not targeted for degradation by CRL4. After UV damage, IP6K1 dissociates from CRL4^DDB2^ and synthesizes the negatively-charged inositol pyrophosphate molecule IP7 that is thought to promote CSN dissociation. Additional changes to the CRL architecture could be required as the CSN can be displaced from CRL^DDB2^ and CRL^CSA^ upon binding, in a neddylation independent manner [96,123]. After the CSN dissociates, neddylation of CRL4 occurs the ligase is activated. Over the course of repair after initial damage, CSN re-associates with the DDB complex, which suggests deactivation of CRL4^DDB2^ [45]. The deactivation process for CRL4 is unclear; however, DDB2 is autoubiquitylated and degraded during its deactivation [124,146]. Conversely, higher levels of the CSN were associated with CSA after UV irradiation, indicating a different regulatory mechanism in TC-NER [45]. Taken together, it suggests that proper timing of CSN dissociation and association with CRL4 is important for functioning NER.

The interplay between the CSN and NER has only been partially elucidated and new insights may come from cellular studies of CSN subunit behaviour *in vivo*. For example, after treating mammalian cell lines with UV, Fuzesi-Levi and colleagues [37] noticed a dose-dependent temporal shuttling of fluorescence-tagged CSN subunits into the nucleus, which was reversed 4 h post-irradiation. Nucleoplasmic and chromatin-associated fractions contained increased levels of CSN protein, while the cytoplasmic fraction did not see a significant change after UV treatment. In addition, they also found that CSN subunits are phosphorylated early after post UV irradiation, and that the level of phosphorylation varies in each of the different cellular fractions. While the phosphorylation sites of CSN1, CSN3 and CSN8 have been mapped, it is not known how these phosphorylation events, or the shuttling of CSN subunits regulate the NER

### 2.6. DNA Double Strand Break Repair

Ionizing radiation (IR, e.g., X-rays) and some chemotherapeutic drugs such as mitomycin C [147], and cisplatin [148] frequently cause DNA strand breaks. Cellular signaling in response to DNA double strand breaks (DSBs) is mediated by proteins in the phosphatidylinositol 3-kinase-like protein kinase family (PIKKs), which include ATM, ATR, and DNA-PK, and by proteins in the poly(ADP-ribose) polymerase (PARP) family[149]. DNA DSBs are repaired by mechanisms that fall under two broad categories: Non-homologous end-joining (NHEJ), and Homologous recombination (HR) (See [150,151,152,153,154] for comprehensive reviews of these pathways). NHEJ is characterized by the ligation of two DSB ends, and is generally considered mutagenic since little to no sequence homology is used for repair [155]. In some situations, there is 5' to 3' end resection, producing short 3' end ssDNA overhangs. These overhangs can anneal to facilitate repair, termed microhomology-mediated end joining (MMEJ), also known as alternative NHEJ [156,157]. Conversely, HR is characterized by the use of long homologous sequences on the undamaged sister chromatid as a template to repair the broken strand, involves more significant end-processing and is less error-prone [158]. During HR, the exposed ssDNA is bound to and stabilized by RPA. Strand invasion of the homologous sequence in the sister chromatid by the ssDNA is then facilitated by RAD51 [159]. Several subtypes of HR have been described [152]. Furthermore, the requirement for an undamaged DNA template restricts HR to the S- and G2-phase of the cell cycle, whereas NHEJ can occur throughout the cell cycle [160,161].

Before a DNA DSB can be repaired, it must be detected, and more than one DSB sensor has been identified in human cells. One DSB sensor is the MRN complex, composed of meiotic recombination 11 (MRE11), RAD50 and nibrin (NBN), which has DNA binding, exonuclease, and endouclease activity [162,163,164]. MRN together with retinoblastoma binding protein 8 (RBBP8, also known as CtIP) stabilizes the DNA ends and promotes initial DNA end resection and HR [165,166]. Thus, CtIP via its role in end resection is thought to control HR as well as checkpoint signaling. Another DSB sensor is the Ku heterodimer formed by the binding of Ku70 (XRCC6) and Ku80 (XRCC5). Ku is a DNA-binding protein that quickly binds to free DNA ends and holds them in close proximity. Ku is important for recruiting proteins in NHEJ [151]. The PARP family members PARP1 and PARP2 are additional DNA damage sensors that recognize single-strand and double-strand DNA breaks, and initiates single-strand break repair and alternative NHEJ [167,168,169]. Why one sensor is preferentially recruited to a DSB site *versus* another (therefore promoting one repair pathway over another) is poorly understood and under intense study, but cell cycle status, nuclear position, and chromatin structure play important roles in repair pathway choice [161,170,171].

As described briefly above, serine-threonine kinases of the PIKK family ATM, ataxia telangiectasic and Rad3 related (ATR), and DNA-PK potentiate the DNA damage signal throughout the cell to coordinate repair. ATM and DNA-PK primarily respond to DNA DSBs, the former through interacting with NBN in the MRN complex [172,173], and the latter through Ku-mediated DNA binding in NHEJ [174]. ATR is activated by the ssDNA binding protein RPA as a result of DNA end resection during DSB repair, or from replication stress [175]. PIKK members also phosphorylate effector proteins, which regulate cell cycle checkpoints, transcription, senescence, and apoptosis [175]. ATM phosphorylates and activates checkpoint kinase 2 (CHEK2; also known as CHK2), a known regulator of the G1/S checkpoint; while ATR targets the related checkpoint kinase 1 (CHEK1; also known as CHK1), a known regulator the intra-S and G2/M checkpoints [176].

Another feature found early in DSB repair is the phosphorylation of H2AX on serine 139 (γH2AX). H2AX is phosphorylated by ATM in response to DSBs but is also targeted by ATR and DNA-PK [177,178]. γH2AX signaling is sustained by the recruitment of mediator of DNA damage checkpoint protein 1 (MDC1), which amplifies the phosphorylation signal and prevents H2AX dephosphorylation [179]. γH2AX and MDC1 also recruit additional mediators to the site, such as tumor suppressor p53-binding protein 1 (53BP1), to the foci [175].

### 2.7. Neddylation in DSB Repair

A clear indication that neddylation is important for DSB repair is that inhibiting this pathway sensitizes cells to IR [180] and that NEDD8 localizes to DNA damage sites [66]. Preliminary studies suggest the possiblity that the STUbL E3 RNF111 interacts with UBE2M to neddylate targets such as histone H4 (H4) at DNA damage sites [66]. In addition, polyneddylated histone H4 is thought to be important for tethering of RNF168 to the DNA DSB site [66]. RNF168 can modify histone H2A as both a NEDD8 and ubiquitin E3 ligase, and its ubiquitinylase activity towards H2A is promoted by its neddylation and inhibited by NEDP1, which deneddylates RNF168 and inhibits its interaction with the ubiquitin E2 enzyme Ubc13 [181]. Neddylation of RNF168 regulates the downstream recruitment of BRCA1 following DNA damage by promoting H2A ubiquitination by RNF168 over H2A neddylation [181]. The degree of neddylation of chromatin and DDR proteins following DNA damage may also affect DNA repair pathway choice. RNF111-mediated neddylation, according to Jimeno *et al.* [158], inhibits DNA end resection involving the BRCA1 binding partner CtIP [165,182], therefore making HR less favourable. Gross inhibition of neddylation in the cell with the drug MLN4924, or knockdown of either RNF111 or UBE2M, promotes HR as evidenced by an increase in RPA foci in the nucleus. However, it should be noted that the STUbL activity of RNF111 ubiquitylates SUMOylated proteins, suggesting the role of RNF111 in the DDR is much more complex and may involves multiple UBL pathways [133,183]. What the neddylated targets of RNF111 are in this case will require additional studies. These studies generally suggest that neddylation inhibits DNA DSB repair by HR. This is perhaps an over simplification as there is also evidence that neddylation differentially affects HR sub-pathways. Specifically, MLN4924 treatment appears to promote single-strand annealing (SSA), presumably by increasing DNA end resection, but inhibits HR by gene conversion [158]. Initial studies also suggest that neddylation could be important for terminating DNA repair. For examples, one study found that inhibiting neddylation with MLN4924 delayed the release of NHEJ factors, such as Ku, from the break site after repair, possibly indicating that the dissolution of NHEJ factors from DNA breaks occurs through CRL-mediated ubiquitylation [184].

### 2.8. CSN in DSB Repair

The CSN appears be important for proper regulation of DNA double-strand break (DSB) repair response. Loss of CSN5 increased DSB defects and sensitized cells to DNA damage [185]. This is accompanied by increased γH2AX, and activation of cell cycle checkpoint proteins. In addition, both ATM and ATR mediated effects are increased in response to CSN5 knockdown [14,148]. Since CSN5 harbours the deneddylase enzyme activity of the CSN complex, this data implies a possible role for deneddylation in DNA DSB repair. However, a non-catalytic role for free CSN5 cannot be fully discounted. There is also evidence that the CSN responds to DNA DSBs through changes in the localization and abundance of CSN subunits and/or coordination of the various repair pathways. For example, when HT29 cells were treated with different doses of the DNA damage agent mitomycin C, Feist *et al.* [47] noted a dose-dependent increase of CSN subunits. In addition, the CSN is recruited to DSB sites following IR, and the recruitment depends on neddylation [20,184]. Currently it is unknown if the entire CSN complex, subcomplexes or individual subunits are mediating specific events during DNA DSB repair. However, CSN8 can interact with ATM kinase directly, and CSN3 is phosphorylated on S410 by ATM in response to DNA damage [20]. Mutation of CSN3 (*i.e.*, S401A) to prevent its phosphorylation by ATM can increase radiosensitivity but did not affect the recruitment of CSN3 to DNA DSBs. CSN3 phosphorylation is also required for efficient RAD51 repair foci formation, suggesting a role for CSN3 in end resection and possibly HR [20]. However, despite evidence for neddylation and/or CSN subunits in promoting HR [20,158], how the CSN might regulate DNA repair pathway choice between NHEJ and HR remains unclear. It has been speculated that repair pathway choice may depend on the degree of deneddylation following the initial round of neddylation [158]. Thus echoing the role of ubiquitinylation in DNA repair, it appears that both neddylation and deneddylation are required for the regulation of DNA DSB repair.

## 3. Conclusions

The DNA damage response (DDR) is tightly controlled by reversible protein post-translational modifications by ubiquitin-like (UBL) proteins including NEDD8. Neddylation is a primary regulator of cullin E3 ligase (CRL) activity, which target a number of DDR and cell cycle checkpoint proteins. The evidence that CRLs are transiently at the damage site raises the question of not only their role at the DNA break but also how there are regulated during the DDR. The CSN deneddylase complex is a major regulator of CRL activity, which we have discussed here in the context of the nucleotide excision repair (NER). However, this does not exclude possible roles for the CSN complex (or its subunits) outside of CRL regulation, particularly during DNA double-strand break (DSB) repair. Although an over simplification, neddylation appears to oppose DNA DSB repair by homologous recombination by affecting DNA end resection; however, the substrates of this neddylation and the details of this effect remain poorly characterized. Importantly, due to the intercommunication amongst ubiquitin and other UBL modification pathways including SUMOylation and neddylation, it will be increasingly important to consider crosstalk between these pathways in future studies.

While the CSN is a dynamic complex, not all the subunits have been studied in relation to DNA repair. Furthermore, the regulation of CSN subunits through their phosphorylation during the DDR and the ultimate impact these events have on both the progression of DNA repair and repair pathway choice is only beginning to be understood. In addition, the mechanism for shuttling the CSN to different cellular compartments also remains to be characterized and could be influenced by non-cullin interacting partners. A better understanding of the CSN and neddylation will not only provide a better understanding of the DDR but it may potentially have therapeutic implications for cancer treatment. Many tumors display defects in DDR, making them susceptible to DNA damaging agents [186]. However, some tumors adapt to DNA damage-inducing chemotherapeutic drugs, such as cisplatin, by enhancing DDR activity, precipitating the need for DDR inhibitors [187]. In some human cancers, elevated expression levels of CSN5 and CSN6 are correlated with cancer progression and poor prognosis [148,188,189,190,191], which may be due to increased DDR function reducing the efficacy of cytotoxic therapies employing DNA damaging agents. Given that both the CSN and neddylation appear to have a significant regulatory function in the DDR, we predict that the CSN will be an increasingly attractive drug target for the development of chemotherapy and radiosensitization agents to treat cancer.
